# Exploring genetic counselors' practice of discussing clinical trials with patients

**DOI:** 10.1002/jgc4.1934

**Published:** 2024-06-09

**Authors:** Thea Bloom, Katherine E. Bonini, Melissa Gutierrez‐Kapheim, Lisa M. Kinsley, Maureen E. Smith, Debra Duquette

**Affiliations:** ^1^ Center for Genetic Medicine Northwestern University Chicago Illinois USA; ^2^ Institute for Genomic Health Icahn School of Medicine at Mount Sinai New York New York USA; ^3^ Neurology Department Northwestern Medicine Chicago Illinois USA; ^4^ Present address: Cook County Health Chicago Illinois USA

**Keywords:** attitudes, clinical trials, counseling techniques, genetic counseling, public health, referral practices

## Abstract

Despite concerted and accelerated efforts to increase the knowledge of medicine and disease via clinical studies, clinical trials continue to face low enrollment for all patient groups. The dissemination of the availability of clinical trials to individuals with or at risk for hereditary disorders is critical. This study acts as a foundation in determining an unexplored role of clinical trial discussion in genetic counseling practice. Board‐certified, patient‐facing genetic counselors in the United States were invited to participate in an anonymous survey via the National Society of Genetic Counselors. Between February and April 2022, 157 participants (*N* = 157) completed the survey on clinical trial discussion with patients, barriers, and facilitators to discussing clinical trials with patients, research experience, and demographics. Survey results identified that most respondents have discussed the availability of clinical trials with a patient (85%). Almost one‐third have previous research experience working for a clinical trial (30%). Most agreed that discussions of clinical trials are within the scope of genetic counseling (82%); however, one‐third were not comfortable discussing them with patients (34%). Respondents who know how to find specific clinical trials (*p* < 0.001) were reportedly more likely to be comfortable discussing clinical trials with their patients. In addition to clinical research exposure, this study suggests that further education and training is necessary for genetic counselors to learn how to find and identify specific clinical trials for their patients. In turn, we hope for this to increase genetic counselors' comfort of clinical trial discussion.

**What is known about this topic**

Additional healthcare providers are needed to assist in the dissemination of clinical trial information, as it is a key factor in decision‐making for patients. Current literature describes the practice of other allied healthcare professionals, including physicians, nurses, and nurse practitioners; however, there are currently no published studies describing the practice of patient‐facing genetic counselors in identifying clinical trials for patients who may qualify, or how genetic counselors incorporate this into their clinical practice.

**What this paper adds to the topic**

This study acts as a foundation in determining an unexplored role of clinical trial discussion in genetic counseling practice. As one of the first studies to explore this topic for genetic counselors, these findings suggest our sample population is a group of highly motivated genetic counselors that may be representative of the larger genetic counseling community.

## 1 INTRODUCTION

Clinical trials involve human volunteers that receive a specific intervention planned by researchers. These interventional studies, which include a change in procedure, physical device, or drug treatment such as gene therapy, are critical in improving healthcare, treatment, and understanding of disease (National Institutes of Health, 2019). Yet despite their importance in furthering medical knowledge and increasing healthcare quality, discussions about enrollment to clinical trials via healthcare provider interactions are often limited (Albrecht et al., 2008; Getz, 2017; Shields et al., 2008 and Umscheid et al., 2011). These conversations are crucial since participation is more common for individuals who have learned about clinical trials from their healthcare provider (Baquet et al., 2006). Lack of enrollment is the leading cause of clinical trial failure, and between 2008 and 2017, 55% of all clinical trials failed simply because there were not enough participants (National Academies of Sciences, Engineering, and Medicine, 2022).

Genetic counselors are in an opportunistic situation to share relevant information about clinical trials with their patients. In addition to understanding the research process, they have unique training to communicate with and counsel patients (Accreditation Council for Genetic Counseling, 2019). Although practices may differ between institutions and researchers, typical recruitment steps of clinical trials include the identification of eligible participants, discussion of the trial, conduction of screening in accordance with trial protocol, and enrollment based on eligibility criteria (Chaudhari et al., 2020). Genetic counselors have historically been mediators between the clinical and research worlds, helping patients understand complex issues within genetic research and aid in patient decision‐making, and therefore are poised to explain and invite patients to participate in clinical trials (Markel & Yasher, 2004). Furthermore, the 2019 Accreditation Council for Genetic Counseling (ACGC) practice‐based competencies requires that genetic counselors be able to identify available research opportunities and referrals for patients (Accreditation Council for Genetic Counseling, 2019).

Clinical trials are of importance and relevance to genetic counseling patients. In one study that surveyed the dominantly inherited Alzheimer's network, researchers found that 72% of participants who did not want to know their own genetic status said that they would be willing to have genetic testing if it allowed them to participate in a clinical trial of familial Alzheimer's disease (Grill et al., 2015). This study demonstrates how research opportunities can be a strong motivator for genetic testing and further supports why genetic counselors should be discussing them with their patients.

As developments in genomic medicine, especially in gene therapy, continue to grow, clinical trials prove to be especially relevant for patients seeking genetic testing. In a 2017 report, almost 2600 gene therapy clinical trials had been approved worldwide. Currently, most gene therapy clinical trials focus on cancer (65%), followed by inherited monogenic diseases (11.1%) and cardiovascular disease (6.9%) (Ginn, 2018). In addition to creating new therapies and treatments, the results of molecular testing can be used to reduce study length and size, increase the chance of success, and decrease overall study cost (Gower et al., 2016). Previous studies have identified the use of stratified medicine using genetic modifiers to improve clinical trial power for rare diseases and populations with a limited sample size available (Mead et al., 2016). As more clinical trials consider genomic‐based stratification in the era of personalized medicine, the role of genetic counselors may continue to grow as an important facilitator in clinical trials (Illes et al., 2019).

There are currently no published studies focused on describing the practice of genetic counselors in identifying clinical trials for patients who may qualify for them, or how genetic counselors incorporate discussion of clinical trials with patients into their clinical practice. In this study, we explore if and how genetic counselors discuss clinical trials in practice. Specifically, we aimed to (1) determine if patient‐facing genetic counselors are disseminating the availability of clinical trials to their patients and if certain genetic counseling specialties (e.g., oncology, pediatric, neurology, cardiology) discuss them more than others and (2) to identify real and perceived barriers and facilitators to dissemination of clinical trials in a clinical genetic counseling session.

## 2 METHODS

This study was granted an exemption by the Northwestern University Institutional Review Board. Implied informed consent was obtained for individuals who voluntarily completed the online survey and submitted their responses.

### 2.1 Participants & recruitment

Study participants were board‐certified, patient‐facing genetic counselors in the United States, working in all specialties within clinical care. An electronic survey invitation was distributed through the National Society of Genetic Counselors' student research listserv, which is sent to approximately 4500 NSGC members. Two emails were sent throughout the recruitment process, including a reminder email sent 2 weeks prior to survey closure. No previous experience with clinical trials or research was required or mentioned in the survey invitation. Data were collected anonymously between February and April 2022.

### 2.2 Procedure and survey design

A survey was designed by the researchers to explore how participants discuss clinical trials with their patients (Appendix S1). At the beginning of the survey, participants were asked if they work in clinical care. If yes, this question was followed by the amount of time spent with patients in their current practice. Only participants that selected that they work in direct patient care either “full‐time patient facing” or “part‐time patient facing” were included in the analysis. Participants were provided the NIH definition of clinical trials in order to understand and distinguish clinical trials from observational studies (NIH, 2019). The survey consisted of four sections: (1) demographics I, (2) barriers and facilitators to discussion of clinical trials with patients, (3) discussion of clinical trials with patients and research experience (including if participants currently work within any research role at their institution), and (4) demographics II. In demographics I, participants were asked about their specialty, time spent with patients, years practiced, work setting, and whether their clinic is considered a safety‐net hospital. In demographics II, participants were asked their race and gender identity. Barriers and facilitators to the discussion of clinical trials with patients were assessed using statements followed by a Likert scale. In the third section, participants were asked about whether they discuss clinical trials with patients, with how many patients, and their prior research roles. An optional free‐text response was provided for participants to describe what would make discussing clinical trials easier. Questions were created by the researchers as well as adapted from the following resources: the National Society of Genetic Counselors' Professional Status Survey (demographics), Simons Searchlight (time limitations as a barrier), Denver Health LGBT Health Services (demographics), and Versta Research (demographics). See Supplemental D for further details. After completing the survey, participants had the option to provide their email via an anonymized form to enter a raffle for one of five $100 Visa gift cards.

### 2.3 Data analysis

The survey data were collected from Qualtrics software, Version 1.2022 of Qualtrics and analyzed in IBM SPSS Statistics for Windows, Version 28.0. Unfinished surveys were not included in the final analysis due to lack of critical information about clinical trial discussion practice. Descriptive statistics were used to assess barriers and facilitators of clinical trial discussion with patients. Frequencies were calculated for each survey question. Chi‐square and Fisher's exact tests were used for select questions to compare participant characteristics with clinical trial discussion and Likert scale facilitator and barrier statements. For *p*‐value analysis, Likert scale survey responses were collapsed into two categories: strongly agree/agree and strongly disagree/disagree. Open‐ended response text was not analyzed.

## 3 RESULTS

One hundred and fifty‐seven participants met study criteria and completed all or most of the survey (Appendix S2). Participant demographics are listed in Table 1. Most participants were female (*n* = 144, 92%) and self‐described as White (*n* = 141, 90%). The most common specialties of participants were cancer (*n* = 49, 31%), pediatric (*n* = 41, 26%), and prenatal (*n* = 29, 18%). Most participants have practiced as a genetic counselor between 1 and 10 years (cumulative *n* = 134, 72%) and in current practice at an academic medical center (*n* = 92, 59%). Most participants do not currently work in a research role at their clinic or institution (e.g., involvement with student thesis projects or Institutional Review Boards) (*n* = 124, 80%) or have not worked in a research role in the past as a genetic counselor (*n* = 110, 79%). When asked if their clinic is considered a safety‐net hospital, 36% said yes (*n* = 56) and over one‐third was not sure (*n* = 54, 34%).

**TABLE 1:** Participant demographics (*N* = 157).

Characteristic	Number	%
Specialty (*n* = 157)		
Cancer	49	31
Pediatric	41	26
Prenatal	29	18
Neurogenetics	11	7
General/adult	5	3
Ophthalmology	5	3
Other^a^	17	11
Years practiced (*n* = 157)		
0–2 years	61	39
3–5 years	39	25
6–10 years	34	22
11–30+ years	23	15
Primary work setting (*n* = 156)		
Academic medical center	92	59
Private hospital or clinic	36	23
Community hospital	20	13
Other^b^	8	5
Self‐reported race^c^ (*n* = 156)		
White	141	90
Asian or Pacific Islander	12	8
Multiracial or biracial	4	3
Black or African American	2	1
Native American or Alaskan Native	1	1
Other (Responses included: Southwest Asian and Middle Eastern)	3	2
Self‐reported Ethnicity (*n* = 157)		
Non‐Hispanic	151	96
Hispanic/Latinx	4	3
Prefer not to respond	2	1
Gender Identity (*n* = 157)		
Woman	144	92
Man	7	5
Non‐binary/genderqueer/gender fluid	4	3
Prefer not to respond	2	1

^a^ Includes ART/IVF, cardiology, consumer/personal genomics, genomic medicine, metabolic, mitochondrial, nephrology, and skeletal dysplasias.

^b^ Includes laboratory, organization or agency, self‐employed.

^c^ n is greater than 156 as more than once choice possible.

### 3.1 Clinical trial discussion

Most participants discuss the availability of clinical trials with a patient in their current practice (83%) and 85% have discussed it in past or current practice (see Table 2). When asked to estimate the percentage of patients with which clinical trials were discussed within the past year, almost half (48%) reported 1%–10% of their patients. Of the participants who discussed clinical trials with patients, 50% reported themselves as typically initiating conversations about clinical trial availability (50%), followed by physicians (27%); patients (9%); relative, guardian, or caregiver (2%), other allied health profession (2%), or other (11%). Furthermore, 14% of participants reported that discussing clinical trials is included as part of their job description.

**TABLE 2:** Barriers and facilitators of clinical trial discussion (N = 157).

Barriers and facilitators of clinical trial discussion (*N* = 157)	Strongly agree/agree (%)	Strongly disagree/disagree (%)
Barrier statements		
Time limitations within a genetic counseling session prevent me from discussing clinical trials.	32	68
Time limitations outside a genetic counseling session prevent me from discussing clinical trials.	51	49
Discussion of clinical trials is outside of the genetic counseling scope.	18	83
Facilitator statements		
Discussion of clinical trials is relevant to my genetic counseling patients.	76	25
I am comfortable discussing clinical trials with my patients.	66	35
I know how to find specific clinical trials for my patients.	81	20
The institution I work at emphasizes research opportunities to patients.	75	26
The team I work with emphasizes research within our institution.	73	27

Almost all participants have visited the website ClinicalTrials.gov (97%); 83% visited it during their current genetic counseling practice, and 74% in graduate training. When asked in free response what would make it easier to discuss clinical trials with patients, many participants responded about increasing ease of finding a study (e.g., “Information about how to find clinical trials and how to enroll patients in them” and “Better training on how to search trials as well as understand specific language in the postings such as enrollment criteria and patient contact options”).

### 3.2 Previous research and clinical trial experiences

Almost one‐third of participants have previous experience working for a clinical trial (n = 47, 30%) (see Table 3). Of the participants who reported experience working for a clinical trial, 40% described their role as a genetic counselor for patients or participants, 28% as a study coordinator, 9% as an associate investigator, 2% as a principal investigator, and 21% as another role.

**TABLE 3:** Comfortability discussing clinical trials and having prior clinical trial research experience genetic counseling specialty.

Specialty	Comfortable discussing CTs, *n* (%)	Prior CT research experience, *n* (%)
Cancer (*n* = 49)	24 (49)	13 (27)
Pediatric (*n* = 39)	29 (74)	7 (18)
Prenatal (*n* = 29)	17 (59)	8 (28)
Neurology (*n* = 11)	9 (82)	5 (46)
General/adult (*n* = 5)	5 (100)	2 (40)
Ophthalmology (*n* = 5)	5 (100)	2 (40)
Other^a^ (*n* = 19)	14 (74)	10 (53)

Abbreviations: CTs, Clinical trials.

^a^ Other specialties include assisted reproductive technology/in vitro fertilization, cardiology, consumer/personal genomics, genomic medicine, metabolic, mitochondrial, nephrology, and skeletal dysplasia.

### 3.3 Barriers and facilitators of clinical trial discussion

Most participants agreed that discussion of clinical trials is within the scope of genetic counseling. However, one‐third of participants (34%) reported that they were not comfortable discussing clinical trials (see Table 2). Participants who knew how to find specific clinical trials were more comfortable discussing clinical trials with patients (X^2^ (19, *N* = 157) = 39.4, *p* < 0.001). There were no significant associations found between comfort discussing clinical trials and genetic counseling specialty, years practiced, or work setting.

Most participants disagreed that time limitations within a genetic counseling session prevented them from discussing clinical trials with patients (68%). About half of participants (51%) agreed that time limitations outside a genetic counseling session, including having enough time to find studies, prevented them from discussing clinical trials.

### 3.4 Genetic counseling specialty and comfort discussing clinical trials

Different genetic counseling specialties reported more comfort discussing clinical trials (see Table 3). Over half (*n* = 25, 51%) of those working in the cancer specialty reported being uncomfortable discussing clinical trials with their patients. Of participants in the cancer specialty, 27% (*n* = 13) reported previous experience working in research for a clinical trial. Excluding the “Other” category, the specialties that reported the greatest amount of prior clinical trial research experience (neurology, general/adult, and ophthalmology) were also the specialties that reported being the most comfortable discussing clinical trials with patients.

## 4 DISCUSSION

Due to their training in communicating complex topics and value of research and informed decision‐making, genetic counselors are in a unique position to help increase patient awareness of clinical trials and potential research participation. To our knowledge, this is the first study to specifically investigate the experiences, practices, and attitudes of patient‐facing genetic counselors in all specialties and how they use clinical trials in their practice.

While genetic counselors with previous clinical trial research experience were not statistically more comfortable discussing clinical trials (*p* = 0.05804), previous studies among other healthcare professionals have shown a relationship between these variables. It is possible that a larger dataset may have greater power to detect this effect. In a study that surveyed 417 nurses at a comprehensive cancer center, research nurses were predicted to have a higher positive attitude score about clinical trials than other nurses (Burnett et al., 2001). In a qualitative study of specialty nurses in the United Kingdom, positive personal and professional opinions of research were found to be a facilitator for inviting patients to participate in clinical studies (French & Stavropoulo, 2016). Another study of 2400 oncology specialists found a significant correlation between increased number of clinical trial referrals and understanding of clinical trial barriers (Kaplan et al., 2013). A study that surveyed 200 physicians located near NIH Parkinson's disease network clinics suggested that increasing medical student exposure to research may increase their familiarity and comfort with research as practicing physicians (Mainous et al., 2008). Similarly, our study suggests that previous research experience may positively impact clinician comfort with discussing clinical trials with patients. Exposure to clinical research throughout graduate training, including rotations, fieldwork, and internships, may be beneficial for genetic counselors and their practice.

Another important finding of our study identified that participants who reported knowing how to find specific clinical trials were more comfortable discussing them with their patients. Similar to our findings, French and Stavropoulo (2016) found that nurses were less likely to invite patients to clinical research when they did not understand clinical trial availability and details, including study design or purpose. Since genetic counselors primarily use ClinicalTrials.gov to find studies, it is possible that genetic counselors are unclear about how to find specific details including study design and purpose. Although all registered clinical trials are listed on ClinicalTrials.gov, studies have shown that the registry often lacks critical information, including detailed study descriptions, institution and contact information, and eligibility criteria (Borysowski et al., 2021). In addition, available study descriptions are often difficult to understand: on average requiring 18 years of education, according to readability assessment algorithms (Wu et al., 2016). This aligns with free‐text responses from some of our participants, which stated that ease of finding clinical trials would help facilitate discussion of them with patients.

There are a number of potential barriers to discussion of clinical trials by genetic counselors that were explored in this study. Similar to our study, a survey of 455 Pennsylvania nurse practitioners found that participants were less likely to bring up the topic of clinical trials with patients if they were not comfortable discussing the options of entering a clinical trial with patients (Ulrich et al., 2011). Ulrich et al. also found that participants desired more information regarding available studies. In a previous study of over 2000 physicians and nurses, reasons for not referring patients included lack of access to clinical trial information, not enough time to research and learn about a clinical trial, lack of time to discuss the clinical trial with patients, and uncertainty of where to refer patients (Getz, 2017). Several of these findings reflect the responses of our participants. However, in contrast, our participants felt they had enough time in a session to discuss clinical trials with patients. Increasing genetic counselors' ability to find studies effectively and in a time‐efficient manner may help decrease the amount of time needed by genetic counselors to locate clinical trials for their patients.

Advocacy organizations, including Facing Hereditary Cancer Empowered (FORCE), have worked to increase education and clinical trial understanding through research pages and clinical trial workshops for genetic counseling students and NSGC members, including how to identify specific clinical trials available for individuals with a pathogenic variant. This was prompted by a recent needs assessment survey regarding support resources for young and metastatic breast cancer patients, in which the majority of genetic counselor respondents reported that they had never referred these patients to a clinical trial in their practice (Clark et al., 2023). The authors found that genetic counselor familiarity with clinical trials, however, increased with years of experience. This suggests that training of genetic counselors to identify appropriate clinical trials, coupled with mentorship from more experienced clinical genetic counselors, may improve patient access to clinical trials. Genetic counseling graduate training programs, such as the University of South Carolina School of Medicine's Genetic Counseling Program, have developed rotations for genetic counseling students to learn about clinical research and the process of clinical trial participation (Greenwood Genetic Center, 2024). Further education and training is necessary for genetic counselors to learn how to find and identify specific clinical trials for their patients, and in turn, hopefully increase comfort of clinical trial discussion.

Finally, our findings suggest that further research is needed to determine if specific genetic counseling specialties are more likely to benefit from exposure to clinical trials, including prior clinical trial research experience. Certain genetic counseling specialties, including ophthalmology and neurology, have made rapid progress over the past few decades, including the first gene therapy clinical trial for Leber's congenital amaurosis in 2007 (Uthra et al., 2009). Clinical trials for neurodegenerative diseases have also seen an increase in trial growth, with 413 trials for Alzheimer's disease‐modifying drugs between 2002 and 2012 (Leonard, 2020). Additionally, disease‐modifying drugs for Parkinson's disease that were seemingly promising in phase II failed to be successful in phase III, creating an increased urgency and awareness for the need of genome‐based clinical trials or pre‐trial or post‐trial genetic adjustment (Leonard, 2020). Further research is needed to understand if genetic counselors in these specialties are more comfortable discussing clinical trials due to the accelerated research.

### 4.1 Study strengths and limitations

This is one of the first studies to explore the discussion of clinical trials between genetic counselors and their patients, with a wide variety of patient‐facing genetic counseling specialties represented. As an exploratory study, it brings greater understanding to a previously unpublished topic and identifies how genetic counselors are discussing clinical trials within their institution, including characteristics and experiences that may aid with these discussions. It demonstrates the value genetic counselors with research experience can provide to patient care. Additionally, it identifies where educational resources can be added to increase genetic counselors' comfort discussing clinical trials.

This is an exploratory study, and thus, the findings should be interpreted as exploratory. Additionally, convenience sampling was used to obtain survey responses. However, this is one of the first studies to examine genetic counselor views on this topic, and thus, it still offers important information for future studies to build upon. Limitations of this study additionally include a limited response rate and study sample, with some specialties only consisting of five participants, not allowing for comparison between groups. While reported race, ethnicity, and gender demographics of this cohort were generally representative of the 2022 Professional Status Survey (PSS), certain genetic counseling specialties were overrepresented (pediatric, neurogenetics, and ophthalmology) and underrepresented (cancer and prenatal). Additionally, ascertainment bias is possible within the study sample. Further research is necessary to understand if the research experiences of this study sample are representative of the larger genetic counseling community. According to the 2022 Professional Status Survey (PSS), 50% of genetic counseling respondents reported being involved in research activities (National Society of Genetic Counselors, 2022). These activities included serving on thesis committees, writing grant proposals, and involvement with institutional review boards. Experience working with clinical trials and research studies was not reported by the PSS. This question may be worth further exploring on the PSS to understand the research experience of the current genetic counseling field.

Additional research is needed to further understand barriers and facilitators of clinical trial discussion between genetic counselors and patients, including a qualitative study of genetic counselors' experiences with clinical trials. This may also include a further analysis of the open‐ended responses provided by participants in this survey. Both may allow for further exploration of the reasoning and context for how and why genetic counselors discuss clinical trials with their patients. In addition, further comparison of attitudes among and between specific genetic counseling specialties may be useful, including comparison of specialties with accelerated research.

## 5 CONCLUSION

This is one of the first studies to investigate the clinical trials experiences, practices, and attitudes of patient‐facing genetic counselors. In this study, almost one‐third of participants report experience working in research for a clinical trial. Most participants agree that discussions of clinical trials are within the scope of genetic counseling; however, one‐third are not comfortable discussing them with patients. Genetic counselors who know how to find specific clinical trials and genetic counselors with previous research experience working for a clinical trial are more likely to be comfortable discussing clinical trials with their patients. Increasing research experiences as well as providing educational opportunities and workshops about clinical trials may help genetic counselors become more comfortable discussing research studies with patients. This key role in identifying and discussing appropriate research studies with patients is described as a 2019 ACGC practice‐based competency. With a distinctive skillset to explain complex processes and help patients through difficult decisions, in addition to valuing the research process, genetic counselors can and should be familiar with clinical trial studies.

## AUTHOR CONTRIBUTIONS

Author Debra Duquette confirms that she had full access to all the data in the study and takes responsibility for the integrity of the data and the accuracy of the data analysis. All of the authors gave final approval of this version to be published and agreed to be accountable for all aspects of the work in ensuring that questions related to the accuracy or integrity of any part of the work are appropriately investigated and resolved. Author contributions are as follows: Thea Bloom was involved in conceptualization; data curation; investigation; formal analysis; visualization; methodology; writing—original draft; writing—reviewing & editing. Katherine E. Bonini was involved in conceptualization; methodology; project administration; supervision; visualization; writing—original draft; writing—reviewing & editing. Melissa Gutierrez‐Kapheim was involved in formal analysis; methodology; validation; writing—reviewing & editing. Lisa M. Kinsley was involved in writing—reviewing & editing. Maureen E. Smith was involved in methodology, writing—reviewing & editing. Debra Duquette was involved in conceptualization; methodology; project administration; supervision; writing—original draft; writing—reviewing & editing.

## CONFLICT OF INTEREST STATEMENT

Debra Duquette is a paid consultant for the Centers for Disease Control & Prevention, Division of Cancer Prevention & Control and is also a paid member of the AIM Genetic Testing Panel. Maureen E. Smith is an associate editor for the Journal of Genetic Counseling. Thea Bloom, Katherine E. Bonini, Melissa Gutierrez Kapheim, and Lisa M. Kinsley have no conflicts of interest to disclose.

## ETHICS STATEMENT

Human Studies and Informed Consent: This study was reviewed and granted an exemption by the Northwestern University Institutional Review Board. All procedures followed were in accordance with the ethical standards of the responsible committee on human experimentation (institutional and national) and with the Helsinki Declaration of 1975, as revised in 2000. Implied informed consent was obtained for individuals who voluntarily completed the online survey and submitted their responses.

Animal Studies: No non‐human animal studies were carried out by the authors for this article.

## Supporting information


Data S1

## Data Availability

The data that support the findings of this study are available from the corresponding author upon reasonable request.
