# Supplementary material for: Exploring genetic counselors' practice of discussing clinical trials with patients
Source: J Genet Couns. 2024 Jun 9;34(2):e1934. doi: 10.1002/jgc4.1934 (PMC11907176; doi:10.1002/jgc4.1934)
Supplement: Supplementary file 1 — Data S1 [file JGC4-34-0-s001.docx]

### **Supplemental A) Survey**

*Genetic Counselors’ Discussion of Clinical Trials in Practice: Identifying Barriers and Facilitators of Discussion with Patients*

**Title of Research Study:** Genetic Counselors’ Use of Clinical Trials in Practice: Identifying Barriers and Facilitators of Discussion with Patients

**Principal Investigators:** Debra Duquette

**Student Investigator:** Thea Bloom

**Supported by:** This research is supported by the Northwestern University’s Masters Graduate Program in Genetic Counseling.

**Information about this research study:**

The purpose of this study is to explore practicing genetic counselors’ discussion of clinical trials in clinical practice with patients. You asked to complete an anonymous questionnaire, that should take approximately 10 – 15 minutes to complete. There are no known risks for participating in this study.

**Who can participate in this survey?**

You are eligible for this study if you are a board-certified, patient-facing genetic counselor in the United States.

**Is this study voluntary?**

At any point throughout the survey, you may choose to exit the survey. Your decision participate in this study is voluntary. All responses are confidential. Names are not asked in this survey and will not be used in publication or research data.

**Are there any incentives for participation in this study?**

As an incentive to participate, you can choose to enter a raffle to receive 1 of 5 $100 Visa gift cards via email. Interested participants may securely provide their email address separately from survey responses to allow for anonymity.

This study was approved by the Northwestern University IRB.

If you have questions about this study, please contact Thea Bloom at thea.bloom@northwestern.edu and/or Debra Duquette, MS, CGC at debra.duquette@northwestern.edu.

**I Demographics:**

1. Are you a board-certified genetic counselor?

a. Yes

b. No [END SURVEY]

1. Do you work as a genetic counselor in clinical care?
2. Yes
3. No [END SURVEY]

1. Please describe the amount of time spent with patients in your current practice:

***Note:*** *Throughout this survey, the term “patient” additionally refers to participants, clients, and individuals counseled by the genetic counselor in a genetic counseling session.* *Sessions may occur in the research and/or clinical setting.*

a. Full-time patient-facing

b. Part-time patient-facing

c. Rarely interact with patients

d. Never interact with patients

4. Do you have more than one specialty?

a. Yes

b. No

c. Not applicable

1. Please describe your primary genetic counseling specialty:
   1. Cancer
   2. Cardiology
   3. Consumer Genomics/Personal Genomics
   4. General/Adult
   5. Genomic medicine
   6. Hematology
   7. Metabolic
   8. Molecular/Cytogenetics/Biochemical Testing
   9. Neurogenetics
   10. Newborn screening
   11. Ophthalmology
   12. Pediatric
   13. Pharmacogenetics
   14. Public health
   15. Psychiatric
   16. Preimplantation genetic testing, ART/IVF, Infertility
   17. Prenatal
   18. Other (specify): [free text]

6. In which state do you primarily practice?

(Drop down menu of all states)

7. How would you describe the primary location of your practice setting?

a. Urban

b. Suburban

c. Rural

d. Virtual

e. Other: ________ (free text)

8. Please describe the number of years you have practiced as a genetic counselor:

a. Less than one year

b. 1 – 2 years

c. 3 – 5 years

d. 6 – 10 years

e. 11 – 15 years

f. 16 – 20 years

g. 21 – 30 years

h. Over 30 years

9. What is your primary work setting?

a. Academic medical center

b. Community hospital

c. Private hospital or clinic

d. Laboratory

e. Organization or agency (government, professional, health advocacy, etc.)

f. Insurance company

g. Pharmaceutical company

h. Self-employed

i. Other work setting: (free text)

10. 9a. [If select hospital] Is your clinic considered a safety-net hospital? (Defined as hospitals and other providers that organize and deliver a significant level of health care and other health-related services to patients with no insurance or with Medicaid)^1^

a. Yes

b. No

c. I am not sure

**II Barriers and Facilitators to Discussion of Clinical Trials with Patients**

*Clinical trials, also known as interventional studies, are the use of human volunteers in research to increase the knowledge of medicine. These participants receive a specific intervention planned by the researchers. This may be a physical product, like drug or device, change in procedure, such as diet or other activity, or educational intervention.*

Please select how you feel about each corresponding statement. For each statement, please think generally and consider what is typical for your practice and clinic.

11. Many of my patients qualify for a clinical trial.

a. Strongly agree

b. Agree

c. Disagree

d. Strongly disagree

e. I don’t know if my patients qualify for a clinical trial.

Please provide any details you wish to share about your answer. [Free text]

12. Time-limitations within a genetic counseling session prevent me from discussing clinical trials.

a. Strongly agree

b. Agree

c. Disagree

d. Strongly disagree

Please provide any details you wish to share about your answer. [Free text]

13. Time-limitations outside of the genetic counseling session prevent me from discussing clinical trials (e.g., not enough time to find studies for patients).

a. Strongly agree

b. Agree

c. Disagree

d. Strongly disagree

14. Discussion of clinical trials are relevant to our genetic counseling patients.

a. Strongly agree

b. Agree

c. Disagree

d. Strongly disagree

Please provide any details you wish to share about your answer. [Free text]

15. I am comfortable discussing clinical trials with my patients.

a. Strongly agree

b. Agree

c. Disagree

d. Strongly disagree

Please provide any details you wish to share about your answer. [Free text]

16. Discussion of clinical trials are outside of the genetic counseling scope.

a. Strongly agree

b. Agree

c. Disagree

d. Strongly disagree

Please provide any details you wish to share about your answer. [Free text]

17. At my clinic or institution, clinical trials are discussed with my patients by:

| Designated research coordinator | Strongly agree | Agree | Disagree | Strongly disagree |
| --- | --- | --- | --- | --- |
| Physician | Strongly agree | Agree | Disagree | Strongly disagree |
| Nurse | Strongly agree | Agree | Disagree | Strongly disagree |
| Genetic counselor | Strongly agree | Agree | Disagree | Strongly disagree |
| Other healthcare provider | Strongly agree | Agree | Disagree | Strongly disagree |

Please provide any details you wish to share about your answer. [Free text]

18. I know how to find specific clinical trials for my patients.

a. Strongly agree

b. Agree

c. Disagree

d. Strongly disagree

Please provide any details you wish to share about your answer. [Free text]

19. What would make it easier to discuss clinical trials with your patients?

a. [Free text]

**III Discussion of Clinical Trials with Patients**

20. The institution I work at emphasizes research opportunities to patients.

a. Strongly agree

b. Agree

c. Disagree

d. Strongly disagree

21. The team I work with emphasizes research within our institution.

a. Strongly agree

b. Agree

c. Disagree

d. Strongly disagree

22. Do you have prior experience working in research for a clinical trial?

a. Yes

b. No

22A. [If select Yes Q22] Please describe your role:

a. Principal investigator

b. Associate investigator

c. Study coordinator

d. Genetic counselor for participants or patients

e. Other (please describe): _____ (free text)

23. Please select the following that best describes you:

| I currently work in a research role at my clinic/institution | Yes | No |
| --- | --- | --- |
| I have worked in a research role in the past as a genetic counselor | Yes | No |
| I have worked in a research role prior to my certification as a genetic counselor | Yes | No |

Please provide any details you wish to share about your answer. [Free text]

24. Have you ever discussed the availability of a clinical trial with a patient (in past or current practice)?

a. Yes

b. No

24A. [If select Yes to Question 25] Do you currently discuss the availability of a clinical trial with patients in your current practice?

a. Yes

b. No

24B. [ If select Yes to Question 25] What party typically initiates the conversation regarding the availability of a clinical trial?

a. You (Genetic counselor)

b. Patient

C. Relative, guardian or caregiver

c. Physician

d. Allied health professional

e. Other (specify): [free text]

24C. [If select Yes to Question 25] Is discussing clinical trials at your clinic or institution included as part of your job description?

a. Yes

b. No

c. Other (specify): [free text]

24D. [If select Yes to Question 25] For the clinical trials that you discuss, are they conducted, recruited, or funded by your institution or clinic?

| Conducted at my institution | Yes | No | I don’t know |
| --- | --- | --- | --- |
| Recruited at my institution | Yes | No | I don’t know |
| Funded at my institution | Yes | No | I don’t know |
| Conducted outside my institution | Yes | No | I don’t know |
| Recruited outside my institution | Yes | No | I don’t know |
| Funded outside my institution | Yes | No | I don’t know |
| Other: [free text] | Yes | No | I don’t know |

24E. [If select Yes to Question 28] Please further describe the discussion of clinical trials with patients/participants: (optional)

a. [Free text]

25. Have you learned about clinical trials for your patients through the following?

| Clinicaltrials.gov | Yes | No | [Free text] |
| --- | --- | --- | --- |
| Patient advocacy website | Yes | No | [Free text] |
| NSGC email | Yes | No | [Free text] |
| Email from clinical trial | Yes | No | [Free text] |
| Approached by clinical trial investigator | Yes | No | [Free text] |
| Word of mouth from colleague | Yes | No | [Free text] |
| Internet search engine | Yes | No | [Free text] |
| Research matching site or tool | Yes | No | [Free text] |
| Other [Please specify] |  |  |  |

26. Have you referred a patient to any resource to search for clinical trials themselves? Please describe.

a. Yes

b. No

27. Have you visited the website clinicaltrials.gov?

a. Yes

d. No

27A. [If yes to Question 28] Please mark when you have visited the website ClinicalTrials.gov:

| In my current genetic counseling practice | Yes | No | [Free text] |
| --- | --- | --- | --- |
| In a previous genetic counseling role | Yes | No | [Free text] |
| In a non-genetic counseling role | Yes | No | [Free text] |
| In graduate training | Yes | No | [Free text] |
| Other: |  |  |  |

27B. [If yes to Question 28] Feel free to provide further information on your interaction with the clinicaltrials.gov website:

a. [Optional free text for comment]

28. Please estimate the percentage of patients with which you discuss clinical trials with in the past year:

a. 0%

B. 1-10%

b. 11-25%

c. 26-50%

d. 51-75%

e. 76-99%

G. 100%

f. Other: [free text]

**III Demographics**

Please select the answer that best describes yourself for the following questions.

29. Are you Hispanic or Latinx?

a. Yes

b. No

c. Prefer not to respond

30. Which of the following categories describe your race? Please select all that apply.

a. Asian or Pacific Islander

b. Black or African American

c. Hispanic or Latino

d. Native American or Alaskan Native

e. White or Caucasian

f. Multiracial or Biracial

g. A race/ethnicity not listed here: (Please specify)

31. Please describe your gender identity:

a. Woman

b. Transgender Woman / Trans Feminine

c. Man

d. Transgender Man / Trans Masculine

e. Non-Binary / Genderqueer / Gender Fluid

f. Two Spirit

g. Prefer not to respond

h. Prefer to self-describe: (free text)

### **Supplemental B) Survey Response Rate**

| 177 | Total survey responses between February and April 2022 |
| --- | --- |
| 1 | Respondent who is not a board-certified genetic counselor, not meeting study inclusion criteria |
| 2 | Respondents who are not working in clinical care, not meeting study inclusion criteria. |
| 17 | Unfinished surveys that did not answer clinical trial discussion practice questions, which were considered critical questions in the survey |
| N = 157 | 157 surveys were used for analytical sample |

### **Supplemental C) Data Tables**

**Table 1 Supplemental Data. Clinical Trial Discussion**

| **Clinical Trial Discussion** | **Number** | **Percent** |
| --- | --- | --- |
| Has discussed the availability of a clinical trial with a patient in past or current practice. (n=157) | 133 | 85% |
| Has discussed the availability of a clinical trial with a patient in current practice. (n=132) | 110 | 83% |
| Estimated percentage of patients clinical trials were discussed within the past year: (n=157) | | |
| 0% | 29 | 18% |
| 1-10% | 76 | 48% |
| 11-25% | 31 | 20% |
| 26-50% | 11 | 7% |
| 51-100% | 10 | 6% |

**Table 2 Supplemental Data. Research and Clinical Trial Experience (N=157)**

| **Research and Clinical Trial Experience (N=157)** | **Number** | **Percent** |
| --- | --- | --- |
| Prior Experience Working in Research for a Clinical Trial | 47 | 30% |
| *Prior Research Role (n=47)* |  |  |
| Principal investigator | 1 | 2% |
| Associate investigator | 4 | 9% |
| Study coordinator | 13 | 28% |
| Genetic counselor for patients | 19 | 40% |
| Other | 10 | 21% |
| Currently Works in Research Role (n=156) | 32 | 20% |
| Job Description Includes Discussing Clinical Trials (n=133) | 19 | 14% |
| Has Visited ClinicalTrials.gov (n=156) | 152 | 97% |

### **Supplemental D) Resources for Survey Design**

- Q5) Please describe your primary genetic counseling specialty: (NSGC, 2021)
- Q9) What is your primary work setting? (NSGC, 2021)
- Q10) [If select hospital] Is your clinic considered a safety-net hospital? (Defined as hospitals and other providers that organize and deliver a significant level of health care and other health-related services to patients with no insurance or with Medicaid) (CDC, 2000)
- II Barriers and Facilitators to Discussion of Clinical Trials with Patients
- *Clinical trials, also known as interventional studies, are the use of human volunteers in research to increase the knowledge of medicine. These participants receive a specific intervention planned by the researchers. This may be a physical product, like drug or device, change in procedure, such as diet or other activity, or educational intervention (NIH, 2019).*
- Q16) Time-limitations outside of the genetic counseling session prevent me from discussing clinical trials (e.g., not enough time to find studies for patients). (Inspired by Simons Searchlight survey)
- Q35) Which of the following categories describe your race? Please select all that apply. (Versta Research, 2020)
- Q36) Please describe your gender identity (Denver Prevention Training Center)

References

CDC. Characteristics of Emergency Departments Serving High Volumes of Safety-net Patients: United States, 2000. Series Report 13, Number 155. 23 pp. 2004.

Denver Prevention Training Center and Denver Health LGBT Health Services. A Guide to LGBTQ+ Inclusive Forms. https://denverptc.org/resource.php?id=231

National Society of Genetic Counselors. (2022). NSGC Professional Status Survey. Retrieved from National Society of Genetic Counselors website: https://www.nsgc.org/Policy-Research-and-Publications/Professional-Status-Survey

National Institutes of Health: U.S. National Library of Medicine. (2019, March). Learn About Clinical Studies. https://clinicaltrials.gov/ct2/about-studies/learn.

Versta Research. How to Ask Race and Ethnicity on a Survey. April, 2020. https://verstaresearch.com/newsletters/how-to-ask-race-ethnicity-on-a-survey/
